# St. Mary's on Medical Education

**Published:** 1921-02-19

**Authors:** 


### ST. MARY'S ON MEDICAL EDUCATION.

At first sight, by comparison with the wealth of material displayed by the Middlesex, there may not appear so much to catch the public eye on the St. Mary's stand, but this hospital takes as its aim a demonstration of the more general reforms needed in the medical teaching of to-day. Particularly their object is to establish a professorial chair in Industrial Medicine at St. Mary's, and in that respect they have ample opportunity of demonstrating throughout other industrial exhibits the direct bearing of the improvements upon the health of the workers themselves.

Their great plan for medical teaching is that the best men shall be found and encouraged to devote their whole time to their work in the schools. In the case of industrial medicine the teachers should visit the heads of great industries throughout the land and find out, first hand, what special diseases were afflicting the employés. Research should be systematically undertaken, to the end that industrial diseases should be gradually lessened and overcome. In any case there most urgently must be far better instruction of medical students in this subject. In the old routine relatively little time was devoted to disease from this essential aspect, and St. Mary's are taking an opportune moment to bring the fact to the notice of the industrial world at large.

